# A Manual of Chemistry, General, Medical and Pharmaceutical, Including the Chemistry of the U. S. Pharmacopeia

**Published:** 1907-04

**Authors:** 


**A Manual of Chemistry, General, Medical and Pharmaceutical, including the Chemistry of the U. S. Pharmacopeia.** By **John Attfield,** F.R.S., Professor of Practical Chemistry to the Pharmaceutical Society of Great Britain, 1862-96. Edited by **Leonard Dobbin,** Ph.D., Lecturer on Chemistry in the University of Edinburgh. Nineteenth Edition. 12 mo, pp. 776. Philadelphia and New York: Lea Brothers & Co. 1906. (Price, $2.50).

Lea Brothers & Company has issued a nineteenth edition of Attfield's Chemistry which has been thoroughly revised by Mr. Leonard Dobbin, of the University of Edinburgh, who has brought it up to the last minute in accordance with the most recent pharmacopeias of this country and Great Britain. As a manual of chemistry for medical and pharmacy students it has no equal and the very thoroughness with which the subject has been covered in this edition makes it preeminently the standard work of today in the English language. Attfield's original plan of teaching as well as the chemistry of every substance concerned in pharmacy and medicine, has been followed out carefully in this edition and its completeness is beyond criticism.

